# Intracellular Delivery of mRNA in Adherent and Suspension Cells by Vapor Nanobubble Photoporation

**DOI:** 10.1007/s40820-020-00523-0

**Published:** 2020-09-27

**Authors:** Laurens Raes, Stephan Stremersch, Juan C. Fraire, Toon Brans, Glenn Goetgeluk, Stijn De Munter, Lien Van Hoecke, Rein Verbeke, Jelter Van Hoeck, Ranhua Xiong, Xavier Saelens, Bart Vandekerckhove, Stefaan De Smedt, Koen Raemdonck, Kevin Braeckmans

**Affiliations:** 1 Laboratory of General Biochemistry & Physical Pharmacy, Ghent University, 9000 Ghent, Belgium; 2 Cancer Research Institute Ghent (CRIG), 9000 Ghent, Belgium; 3 Department of Diagnostic Sciences, Ghent University, 9000 Ghent, Belgium; 4 VIB-UGent Center for Medical Biotechnology, 9052 Ghent, Belgium; 5 Department of Biomedical Molecular Biology, Ghent University, 9000 Ghent, Belgium; 6 Department of Biochemistry and Microbiology, Ghent University, 9000 Ghent, Belgium

**Keywords:** Transfection, mRNA, Photoporation, Optoporation, Gold nanoparticles, Vapor nanobubbles

## Abstract

Efficient and safe cell engineering by transfection of nucleic acids remains one of the long-standing hurdles for fundamental biomedical research and many new therapeutic applications, such as CAR T cell-based therapies. mRNA has recently gained increasing attention as a more safe and versatile alternative tool over viral- or DNA transposon-based approaches for the generation of adoptive T cells. However, limitations associated with existing nonviral mRNA delivery approaches hamper progress on genetic engineering of these hard-to-transfect immune cells. In this study, we demonstrate that gold nanoparticle-mediated vapor nanobubble (VNB) photoporation is a promising upcoming physical transfection method capable of delivering mRNA in both adherent and suspension cells. Initial transfection experiments on HeLa cells showed the importance of transfection buffer and cargo concentration, while the technology was furthermore shown to be effective for mRNA delivery in Jurkat T cells with transfection efficiencies up to 45%. Importantly, compared to electroporation, which is the reference technology for nonviral transfection of T cells, a fivefold increase in the number of transfected viable Jurkat T cells was observed. Altogether, our results point toward the use of VNB photoporation as a more gentle and efficient technology for intracellular mRNA delivery in adherent and suspension cells, with promising potential for the future engineering of cells in therapeutic and fundamental research applications.

**Electronic supplementary material:**

The online version of this article (10.1007/s40820-020-00523-0) contains supplementary material, which is available to authorized users.

## Highlights

- Vapor nanobubble (VNB) photoporation represents a promising physical technique for mRNA transfection of adherent and suspension cells.
- A multitude of parameters related to the VNB photoporation procedure were optimized to enable efficient mRNA transfection.
- VNB photoporation was found to yield five times more living, transfected Jurkat T cells as compared to electroporation, i.e., currently the standard nonviral transfection technique for T cells.

**Electronic supplementary material:**

The online version of this article (10.1007/s40820-020-00523-0) contains supplementary material, which is available to authorized users.

## Graphical abstract

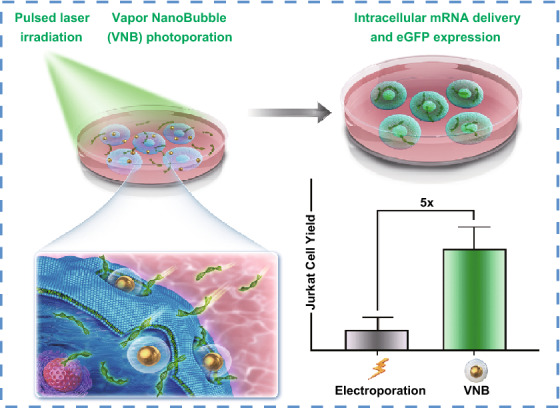

## Introduction

In recent years, mRNA has gained immense interest as a novel class of nucleic acid therapeutics [1–3]. In contrast to DNA therapeutics, mRNA does not require nuclear entry to be functional, being translated instantly after reaching the cell cytoplasm and thus avoiding potential insertional mutagenesis. In addition, mRNA-based therapeutics have a reduced risk of long-term side effects as they are only transiently active inside the cell. The affordability and ease of production have furthermore advanced the development of mRNA as a versatile class of nucleic acid therapeutics, while inherent obstacles such as unfavorable immunogenicity and short half-life time were addressed [1, 2, 4, 5]. Driven by these advances, mRNA has also emerged as a promising tool for ex vivo engineering of adoptive T cells [2]. For this, patient-derived T cells are expanded ex vivo and engineered for targeted cytotoxicity against cancer or viral-infected cells, prior to re-injection into the patient. Recently, the first two chimeric antigen receptor (CAR) T cell products, i.e., Kymriah™ (tisagenlecleucel; Novartis) [6] and Yescarta™ (axicabtagene ciloleucel; Kite Pharma, Gilead) [7, 8], have been approved by the US Food and Drug Administration (FDA) [9, 10]. Genetic modification of the T cells is performed using engineered viruses carrying a vector with the tumor antigen-specific CAR. The use of these viral vectors, however, comes with the limitations of being costly, time-consuming and often having variable results [11–13]. In addition, persistent expression of the CAR construct and risk of insertional mutagenesis contributes to their unfavorable safety profile [11]. While DNA transposons, e.g., sleeping beauty transposon, are considered as a safer nonviral approach, the risk of persistent side effects and insertional mutagenesis remains. mRNA, with its inherent safety features and ease of use, has therefore been raised as a promising alternative over viral- or transposon-based methods for the generation of adoptive T cells [2, 14]. Gene editing by transient Cas9 mRNA expression, for example, became of interest to facilitate highly efficient therapeutic T cell engineering, while reducing the risk of off-target effects and overcoming DNA-related cytotoxicity [15, 16].

The success of mRNA in cell-based immunotherapy strongly relies on the ability to efficiently deliver the mRNA molecules to target immune cells. Of note, improving the efficiency of current transfection technologies is also expected to strongly impact the scalability and production cost of cell-based therapies [5]. Many different technologies have emerged over the years to address the ever recurring issue of intracellular delivery of mRNA, though each of them faces divergent limitations. mRNA is a large negatively charged, single-stranded nucleic acid that can be encapsulated in synthetic nanocarriers for protection against ubiquitous serum nucleases and enhancing endocytic uptake [17]. Gold nanoparticles, for example, have been extensively studied as drug and gene delivery carriers because of their favorable physicochemical properties [18–24]. However, carrier-induced cytotoxicity and low transfection efficiency are common disadvantages for T cells [3]. Physical delivery methods have recently gained attention when it comes to in vitro and ex vivo cell modification, featuring a broad applicability on different cell types and cargos [17, 25]. Electroporation, which makes use of strong electric fields to deliver nucleic acids to the cell interior, is currently the preeminent tool for mRNA transfections of hard-to-transfect immune cells [2, 26]. It should be noted, however, that electroporation was amply shown to come with significant loss of cell viability, induction of unwanted phenotypic changes or loss of cell functionality [17, 27–30]. Laser-assisted photoporation, sometimes also referred to as optoporation, recently came up as a promising gentler technique for intracellular delivery of biological macromolecules [23, 24, 31]. Wayteck et al*.*, for instance, previously showed in a one-on-one comparison between photoporation and electroporation on murine T cells that a threefold higher percentage of siRNA-transfected viable cells was obtained by photoporation as it induced much less cytotoxicity compared to electroporation [32].

In its most straightforward form, photoporation is obtained by focusing high-intensity femtosecond laser pulses onto the cell membrane, thereby inducing very local membrane permeabilization and allowing extracellular molecules to enter the cell cytoplasm [31]. It has been shown to enable efficient mRNA transfection in primary rat neurons even on a subcellular level [33, 34], as well in single neurons of zebrafish embryos [35]*.* Although proven effective for single-cell transfections, its general usability is limited by low-throughput and labor-intensive procedures. The former can, however, be substantially increased by making additional use of photothermal nanoparticles. After attaching to the cell membrane and applying laser irradiation, they can very locally disturb cell membrane integrity. The advantage over traditional photoporation is that these nanoparticles substantially reduce the required light density to enhance membrane permeability, thus allowing to use broad laser beams and resulting in an immensely increased photoporation throughput [36]. In addition, photothermal effects can be efficiently achieved with much less expensive nanosecond pulsed lasers. A particularly effective photothermal phenomenon for creating transient pores in the cell membrane is the generation of vapor nanobubbles (VNBs). These VNBs nucleate from the nanoparticles, such as plasmonic gold nanoparticle (AuNPs), by the rapid evaporation of the immediate surrounding liquid upon pulsed laser irradiation, while heat diffusion to the environment is negligible [37–39]. In addition to AuNPs, graphene-based nanoparticles [40], carbon black nanoparticles [41] and different types of metal alloys [42] have also been suggested for the same purpose. By rapid expansion and subsequent collapse of the VNB after absorption of a laser pulse, high-pressure shockwaves and fluid shear stress can cause physical damage to the neighboring cell membrane structures. In turn, this results in the formation of very localized and transient membrane pores, allowing extracellular cargo to passively diffuse into the cell interior [32, 38, 43, 44]. Conveniently, the technique can be applied to both adherent cells [38] and suspension cells [32, 44], while it is compatible with any type of transparent cell recipient (e.g., culture flasks, multiwell plates). Furthermore, it offers the possibility to transfect even single cells in high throughput [45, 46].

While VNB photoporation has been demonstrated to be suitable to transfect a broad variety of cell types with many different cargos like siRNA [32, 38], nanobodies [40] and other proteins [43], we here report for the first time its suitability for the intracellular delivery of mRNA. Since mRNA is a considerably large (between 20–200 nm), highly negative charged macromolecule compared to smaller antisense oligonucleotides or proteins (between 1–20 nm), effective intracellular delivery of these molecules across the negatively charged cell membrane is particularly challenging [17]. We performed experiments on HeLa and Jurkat T cells as models for adherent and suspension cells. Jurkat T cells serve as a valid model for primary human T cells [47] and are routinely used for screening and optimization of CAR constructs [48–52]. We started by systematically optimizing several parameters related to the VNB photoporation procedure, including AuNP concentration, laser fluence and transfection buffer. We found that for HeLa cells transfection efficiencies up to 38% could be obtained while maintaining a high level of cell viability. In Jurkat T cells, transfection efficiencies up to 20% could be obtained, which could be further enhanced to 45% by applying the procedure up to three times. These results were compared to mRNA transfections by electroporation, which is currently the method of choice for nonviral genetic engineering of T cells. Electroporation appeared to be extremely toxic to Jurkat T cells leading to a reduction by ~ 95% of the metabolic activity of the treated cells, even though in the 5% viable cells very high transfection efficiencies were obtained. Hence, VNB photoporation yielded five times more transfected viable Jurkat T cells as compared to electroporation. Altogether, this study establishes VNB photoporation as a promising, more gentle approach for mRNA transfections of adherent and suspension cells, which is expected to be beneficial for both research and therapeutic purposes.

## Material and Methods

### Materials

Dulbecco’s modified Eagle’s medium containing growth factor F-12 (DMEM/F-12), Roswell Park Memorial Institute 1640 (RPMI-1640), Opti-MEM, L-Glutamine, Dulbecco’s phosphate-buffered saline with Ca^2+^/Mg^2+^ (DPBS+) or without Ca^2+^/Mg^2+^ (DPBS−), penicillin/streptomycin solution (5000 IU/mL penicillin and 5000 μg/mL streptomycin), 0.25% trypsin–EDTA solution, fetal bovine serum (FBS), Hoechst33342, TO-PRO3 iodide (1 mM), CellMask Deep red stain, CellTrace Far Red stain and RNA millennium™ marker were purchased from Life Technologies (Merelbeke, Belgium). CleanCap (cc) enhanced green fluorescent protein (eGFP) and cc Renilla Luciferase (RLuc) mRNA (5′ moU) were received from TriLink Biotechnologies (San Diego, California, USA) and stored at  −  80 °C until use. 60 nm AuNPs were synthesized and coated in-house with the cationic polymer poly(diallyldimethylammonium chloride) (PDDAC), as previously described [24, 43, 44]. Physicochemical properties of the AuNPs were reported before by Raes et al*.*, with a mean zeta potential of + 42 mV, average core diameter of 58 nm and average hydrodynamic size of 113 nm [44].

### In vitro Transcription of MLKL-mRNA

Murine MLKL-encoding mRNA was produced using a pIVTstab-MLKL template, as designed by Van Hoecke et al*.* [53]. The plasmid was first linearized by a PstI restriction digest (Promega, Leiden, the Netherlands), following purification using a QIAquick PCR purification kit (Qiagen, Chatsworth, CA, USA). MLKL-mRNA was obtained by in vitro transcription with the mMESSAGE mMACHINE™ T7 ULTRA Transcription Kit (Life Technologies, Merelbeke, Belgium), according to the manufacturer’s instructions. The in vitro transcribed MLKL-mRNA was eventually purified by LiCl precipitation and stored at − 80 °C until further use.

### Cell Culture

HeLa cells (cervical adenocarcinoma cells, ATCC® CCL-2™) were cultured in DMEM/F-12 supplemented with 10% FBS, 2 mM L-glutamine, and 100 μg/mL penicillin/streptomycin. Cells were seeded at a density of 5 × 10^3^ cells/well of a µ-slide angiogenesis (ibidi GmbH, Martinsried, Germany) and incubated for 24 h at 37 °C, 5% CO_2_ prior to photoporation. µ-slides angiogenesis are compatible with high-resolution microscopy while allowing good attachment of adherent cells thanks to a cell culture-compatible polymer coating. Jurkat E6-1 (human leukemic T cells, ATCC® TIB-152™) and B16F10 cells (murine melanoma cells, ATCC® CRL-6475™) were cultured in RPMI-1640 medium supplemented with 10% FBS, 2 mM L-glutamine and 100 µg/mL penicillin/streptomycin. B16F10 cells were seeded at a density of 25 × 10^3^ cells/well of a 96-well plate and incubated at 37 °C, 5% CO_2_ prior to transfection. Jurkat E6-1 cells were maintained in a humidified atmosphere of 5% CO_2_ at 37 °C, and the culture medium was renewed every 2–4 days. On the day of transfection, 250 × 10^3^ Jurkat E6-1 cells were first incubated with AuNPs, next washed with culture medium (see Sect. 2.5) and eventually transferred to a 96-well plate for photoporation treatment.

### Analysis of mRNA Integrity by Agarose Gel Electrophoresis

mRNA integrity after incubation with HeLa cells, either with or without a prior washing step with Opti-MEM, was assessed by native agarose gel electrophoresis. Prior to addition of the mRNA, the cells were washed once with DPBS, followed by an Opti-MEM washing step of 10 min (only for specified samples). Next, eGFP-mRNA was diluted in Opti-MEM to a final concentration of 0.3 µM and incubated on the cells for the specified time (5, 10, 20, or 30 min). mRNA diluted in Opti-MEM, mRNA incubated with 10 µg/ml RNAseA (Ambion, Merelbeke, Belgium) and a 0.5–10 kb RNA millennium™ marker were taken along as controls. The samples were loaded on a 1% agarose gel, and gel electrophoresis was performed at 100 V for 30 min. For visualization of the mRNA integrity, a Bio-Rad UV transilluminator 2000 (Hercules, CA, USA) was used.

### Visualization and Quantification of AuNP Attachment

Cells were washed once with DPBS (HeLa) or culture medium (Jurkat, 250 × 10^3^ cells) and incubated with AuNP in culture medium for 30 min at 37 °C. Next, the cells were washed once with DPBS (HeLa) or culture medium (Jurkat) and supplemented with new culture medium. AuNP attachment to the cells was visualized by confocal reflection microscopy (C1si or C2, Nikon BeLux, Brussels, Belgium) using a 60× water immersion lens (Plan Apo, NA 1.2, Nikon BeLux, Brussels, Belgium). Jurkat cells were additionally incubated with CellMask deep red (1000×) and Hoechst33342 (1000×) for 10 min at 37 °C to stain the cell membrane and nucleus, respectively. HeLa cells were first incubated with CellTrace Far Red (500×) for 20 min at 37 °C to stain the cytoplasm, after which they were washed twice with culture medium and incubated with Hoechst33342 for 10 min at 37 °C. After staining, the cells were washed with culture medium and imaged using confocal microscopy. Image analysis was performed using the ImageJ software (FIJI, https://Fiji.sc/), including merging the different fluorescent or reflection images into a composite and dilation of the AuNP scattering signal (HeLa), to visualize and quantify the number of cell-attached AuNPs. For each AuNP concentration and each independent experiment, a minimum of 50 (HeLa) or 150 (Jurkat E6-1) cells were analyzed for AuNP attachment by combination of multiple confocal reflection microscopy images recorded for different AuNP incubation samples (≥ 2 wells).

### Determination of the VNB Generation Threshold

A previously reported in-house developed optical setup was used to determine the laser pulse fluence threshold [24, 38], which is defined as the laser fluence of a single laser pulse at which 90% of the irradiated AuNPs generate a VNB. In short, 60 nm AuNPs (stock: ~  4 × 10^10^ AuNPs mL^−1^) were first diluted 50× in ddH_2_O and transferred to a 50 mm γ-irradiated glass bottom dish (MatTek Corporation, Ashland, MA, USA). After sedimentation, the AuNPs sample was mounted on an inverted microscope (TE2000, Nikon BeLux, Brussels, Belgium) and irradiated with a pulsed laser (~  7 ns) tuned at a wavelength of 561 nm (Opolette™ HE 355 LD, OPOTEK Inc, Carlsbad, CA, USA). The laser beam diameter at the sample was 150 µm. The laser pulse energy was monitored using an energy meter (LE, Energy Max-USB/RS sensors, Coherent). An electronic pulse generator (BNC575, Berkeley Nucleonics Corporation) triggered individual laser pulses and synchronized an EMCCD camera (Cascade II: 512, Photometrics) to record dark-field microscopy images before, during and after VNB formation. VNB can be seen distinctly in dark-field microscopy images as brief bright localized flashes of light, due to the increase in light scattering during their lifetime. By quantifying the number of visible VNBs within the laser pulse area (150 µm diameter) for increasing laser pulse fluences, the VNB generation threshold was determined.

### mRNA Transfection by VNB Photoporation

Cells were incubated with AuNPs at different concentrations, as described above. After washing away unbound AuNPs, cells were incubated with Opti-MEM for 10 min as it proved to be beneficial to minimize mRNA degradation. Next, cells were photoporated in the presence of mRNA diluted in the indicated transfection buffer. Opti-MEM, DMEM/F-12, DPBS+  and DPBS were all used as transfection buffer in various experiments. After laser treatment, the cells were supplemented with fresh cell culture medium and allowed to settle for 6 h (RLuc) or 24 h (eGFP) prior to analysis of mRNA expression or cell viability.

### Transfection of Jurkat Cells by Nucleofection

Jurkat cells were transfected with eGFP-mRNA using a 4D-Nucleofector™ according to the manufacturer’s recommendations with the SE Cell line 4D-Nucleofector kit (V4XC-1032) (Lonza, Breda, the Netherlands). First, 2 × 10^5^ Jurkat cells together with 2 µg eGFP-mRNA were resuspended in 20 µL SE cell line solution and transferred to a 16-well Nucleocuvette™ strip. The cells were transfected using the pulse program CL-120 and immediately after supplemented with 80 µL preheated culture medium. Finally, 50 µL of that cell suspension was transferred to a 96-well plate already containing 150 µL preheated culture medium and incubated at 37 °C, 5% CO_2_ for 24 h prior to analysis by confocal microscopy, flow cytometry and viability assays.

### Analysis of eGFP Expression by Confocal Microscopy and Flow Cytometry

Efficiency of eGFP-mRNA transfection was visualized by confocal microscopy (C1si, Nikon BeLux, Brussels, Belgium) using a 10 × objective lens (Plan Apo, NA 0.45) or 60× water immersion lens (Plan Apo, NA 1.2, Nikon BeLux, Brussels, Belgium). Quantification of the percentage eGFP-positive cells was performed by flow cytometry using a CytoFLEX flow cytometer (Beckman Coulter, Suarlée, Belgium). The resulting flow cytometry data were analyzed using FlowJo (Treestar Inc, Ashland, USA) software.

### Analysis of RLuc mRNA Expression

Efficiency of RLuc mRNA expression was determined 6 h after VNB photoporation using the *Renilla-*Glo™ Luciferase assay system (Promega, Leiden, the Netherlands). In short, 50 × 10^3^ Jurkat cells in 50 µL culture medium were combined with an equal volume of *Renilla*-Glo™ Luciferase Assay Reagent. After 10 min, the luminescent signal was measured using a GloMax™ luminometer (Promega, Leiden, the Netherlands). The luminescent signal of each condition was background subtracted (wells with reagent but no cells) and normalized relative to the untreated control.

### CellTiter-Glo® Viability Assay

Viability of HeLa, B16F10 cells or Jurkat cells was assessed for 18 h (B16F10) or 24 h (HeLa, Jurkat) after VNB photoporation or nucleofection using the CellTiter-Glo® luminescent cell viability assay, as recommended by the manufacturer (Promega, Leiden, the Netherlands). Briefly, HeLa, B16F10 and Jurkat cells were supplemented with an equal volume of CellTiter-Glo® reagent for each well, mixed for 5–10 min using an orbital shaker (120 rpm) and transferred to an opaque 96-well plate. After allowing the plate to stabilize for 10 min, the luminescent signal of each well was measured using a GloMax™ luminometer (Promega, Leiden, the Netherlands).

### Evaluation of Cell Viability and Cell Proliferation by Trypan Blue Cell Counting

HeLa cells were first harvested by trypsinization (0.25% trypsin/EDTA), following neutralization with cell culture medium. HeLa or Jurkat cell density in each condition was assessed using a Bürker counting chamber (Brand GMBH + CO KG, Wertheim, Germany) and trypan blue exclusion (0.4%, Sigma-Aldrich, Overijse, Belgium). Cell viability of the different samples was calculated relatively to their respective untreated control. Cell growth was normalized against the untreated control at day 0 and followed for up to 5 days.

### Statistical Analysis

All data are shown as mean ± standard deviation, unless stated differently. Statistical differences were analyzed using the GraphPad Prism 8 software (La Jolla, CA, USA). The statistical tests used in each figure are mentioned in the figure caption. Statistical differences with a *p *value < 0.05 were considered significant.

## Results

### VNB Photoporation Procedure for mRNA Transfection

**Fig. 1:**
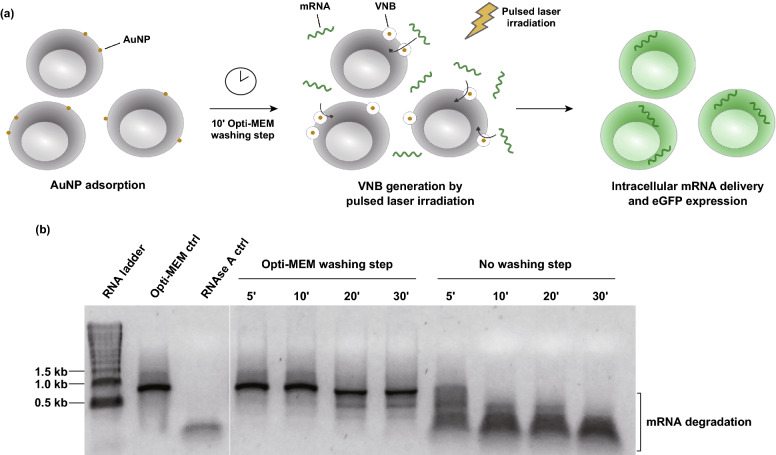
Optimized procedure for mRNA transfection by vapor nanobubble (VNB) photoporation. **a** Schematic illustration of the VNB photoporation procedure for mRNA transfection. **b** Analysis of physical mRNA integrity by native agarose gel electrophoresis; 1 µg eGFP-mRNA was incubated on HeLa cells for the specified amount of time, either with or without a prior washing step with Opti-MEM. As controls, mRNA incubated only in Opti-MEM (Opti-MEM ctrl) or incubated for 30 min with 10 µg mL^−1^ RNAse A (RNAse A ctrl) was included

In this study, we investigated the applicability of VNB photoporation using 60 nm cationic PDDAC-coated AuNPs as photothermal nanoparticles for mRNA transfection. AuNPs with a diameter of 60 nm were previously described to be ideal photothermal sensitizers, requiring a minimum bubble nucleation threshold laser fluence [54]. The experimental procedure for mRNA transfection by VNB photoporation is illustrated in Fig. 1a. For transfection by VNB photoporation, cells are first incubated with cationic AuNPs that will be adsorbed to the cell surface. After washing away unbound AuNPs, irradiation with a single laser pulse (7 ns) leads to the generation of VNBs arising from the cell-bound AuNPs. The inevitable collapse of the VNBs when the thermal energy is consumed causes local pore formation in the cell membrane, allowing extracellular mRNA molecules to diffuse through these membrane pores directly into the cytoplasm. Effective generation of these VNBs can be visualized using dark-field microscopy as a result of an increased amount of light scattering during their lifetime (Fig. S1a). Upon laser irradiation and subsequent VNB generation, the AuNPs are known to fragment into smaller pieces that scatter less light. These AuNP fragments are therefore not visible anymore in the laser-irradiated region [23, 40]. By quantification of the number of generated VNBs within a defined laser irradiated area as a function of the laser fluence (i.e., energy per unit area), the so-called VNB generation threshold fluence was assessed (Fig. S1b). This value is defined as the fluence at which VNBs are formed with 90% certainty and was, in good agreement with previously reported work [44], determined to be 0.9 J cm^−2^.

Considering the inherent labile nature of naked mRNA, premature degradation of these nucleic acids prior to transfection can easily take place. To test this, a native agarose gel electrophoresis assay was performed to qualitatively evaluate the physical integrity of the mRNA after incubation on the cells (Fig. 1b). As we observed that five minutes of incubation of the mRNA solution on HeLa cells already resulted in complete mRNA degradation, an Opti-MEM washing step for 10 min prior to the addition of mRNA to the cells was included to wash away any remaining RNAses as much as possible. After that, the mRNA remained intact for at least 10 min, which is sufficient as the photoporation procedure only lasts ~ 3 min. This washing step was, therefore, included in all further experiments before performing the photoporation procedure.

### mRNA Transfection of Adherent Cells by VNB Photoporation

To date, the applicability of nanoparticle-sensitized photoporation for transfection of mRNA has not yet been investigated. The HeLa human epithelial adenocarcinoma cell line served here as a reference cell type for initial optimization, as it has already previously been used extensively to quantify intracellular delivery of a wide range of molecules (e.g., siRNA and nanobodies) by VNB photoporation [38, 40, 55]. Different parameters related to the VNB photoporation procedure were optimized to reach maximum transfection efficiency with acceptable cytotoxicity, including AuNP concentration, laser fluence, transfection buffer and mRNA concentration. In concordance with the vast majority of scientific studies on adherent cell lines, a cytotoxicity threshold level of 80% was chosen for HeLa cell experiments.

**Fig. 2:**
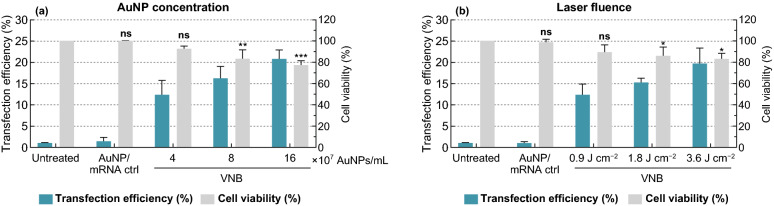
Optimization of gold nanoparticle (AuNP) concentration and laser fluence for eGFP-mRNA transfection in HeLa cells. **a** HeLa cells were photoporated in the presence of 0.3 µM eGFP-mRNA with increasing AuNP concentrations using a fixed laser fluence of 1.8 J cm^−2^. **b** HeLa cells were photoporated in the presence of 0.3 µM eGFP-mRNA with increasing laser fluences using a fixed AuNP concentration of 8 × 10^7^ AuNPs mL^−^. Transfection efficiencies are expressed as the percentage eGFP-positive cells as determined by flow cytometry (*n* ≥ 3). Cell viability values were determined with the CellTiter-Glo assay and expressed relatively to the untreated control (*n* = 3). One-way ANOVAs with Dunnett’s multiple comparison test were performed to determine statistical differences (*ns* = nonsignificant; *p < .05; **p < .01; ***p < .001)

First, different AuNP concentrations and laser fluences were screened for transfection efficiency and cell viability. Cells were incubated for 30 min with AuNP concentrations of 4, 8, and 16 × 10^7^ AuNPs mL^−1^ (Fig. 2a). After washing, this led to ~ 3 ± 1 AuNPs, 5 ± 1 AuNPs and 10 ± 2 AuNPs per cell on average (mean ± SD), as determined by confocal reflection microscopy (Fig. S2). Next, laser irradiation (561 nm) was applied such that every cell in the sample essentially received a single laser pulse of 1.8 J cm^−2^, which is about twice the VNB generation threshold for these gold nanoparticles and therefore ensures effective VNB generation [44]. Using 0.3 µM eGFP-mRNA, up to 21% eGFP-positive cells were obtained depending on the AuNP concentration. At the same time, a slight drop in cell viability was seen 24 h after photoporation, as measured by the CellTiter-Glo assay and further confirmed by a trypan blue cell counting assay (Fig. S3). When considering 20% loss of metabolic activity as a commonly chosen acceptable level of cytotoxicity, 8 × 10^7^ AuNPs mL^−1^ (~ 5 AuNPs/cell) was selected as the most optimal concentration, yielding about 16% eGFP-positive cells. Higher laser fluences were previously suggested to result in bigger VNBs and membrane pores [38] and could therefore further enhance the delivery efficiency of these high molecular weight mRNA molecules. Using the previously optimized AuNP concentration, three different laser fluences were evaluated, *i.e.,* one (0.9 J cm^−2^), twice (1.8 J cm^−2^) and four times (3.6 J cm^−2^) the VNB generation threshold fluence. A moderately increasing trend in eGFP-mRNA transfection efficiency was indeed observed with increasing laser fluences (Figs. 2b and S4), resulting in up to 20% eGFP-positive cells at a laser fluence of 3.6 J cm^−2^, while cell viability remained > 80% for all conditions. Based on these results, it was chosen to continue with an AuNP concentration of 8 × 10^7^ AuNPs mL^−1^ and laser fluence of 3.6 J cm^−2^ for further VNB photoporation experiments on HeLa cells.

**Fig. 3:**
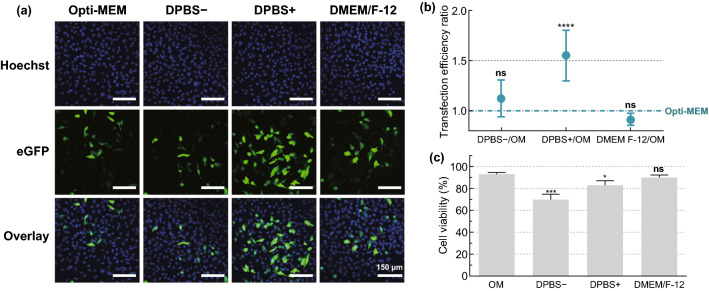
Screening of transfection buffers for mRNA transfection in HeLa cells. HeLa cells were transfected with eGFP-mRNA by VNB photoporation (0.3 µM mRNA; 8 × 10^7^ AuNPs mL^−1^; 3.6 J cm^−2^) using different transfection buffers: Opti-MEM (OM), DPBS−, DPBS+ or DMEM/F-12. **a** Representative confocal microscopy images of HeLa cells 24 h after transfection (Scale bar = 150 µm). **b** Transfection efficiency ratios for different buffers compared to OM, as measured by flow cytometry (*n* = 6). A one-way ANOVA with Dunnett’s multiple comparison test was performed to determine statistical differences between OM and the other buffers (*ns* = nonsignificant; ****p < .0001). **c** Cell viability 24 h post transfection, expressed relatively to the untreated control (*n* = 3). A one-way ANOVA with Dunnett’s multiple comparison test was performed to determine statistical differences between OM and the other buffers (*ns* = nonsignificant; *p < .05; ***p < .001)

Next, we investigated the influence of transfection buffer on eGFP-mRNA transfection efficiency, which reportedly can greatly influence cell viability and transfection efficiency of physical transfection methods [17]. Therefore, we performed photoporation experiments on HeLa cells in different commercially available buffers or media, including Opti-MEM, Dulbecco’s phosphate buffered saline with (DPBS+) or without Ca^2+^/Mg^2+^ (DPBS−) or DMEM/F-12. Confocal microscopy images showed that transfection efficiency was highest for DPBS+ (Fig. 3a). This could be quantitatively confirmed by flow cytometry, with a 1.55-fold increase in the number of transfected cells as compared to Opti-MEM (Fig. 3b), while cell viability remained > 80% (Fig. 3c). Based on these results, DPBS + was selected as transfection buffer for all further transfections of HeLa cells.

**Fig. 4:**
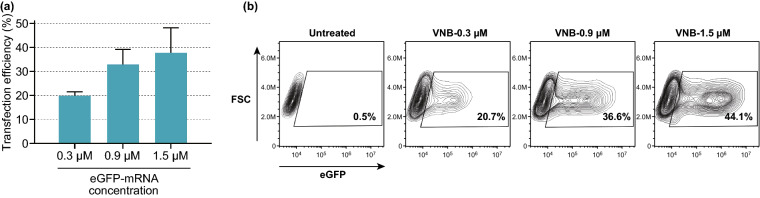
Influence of increasing eGFP-mRNA concentrations on transfection efficiency. **a** Transfection efficiencies, expressed as the percentage eGFP-positive cells, for different concentrations of eGFP-mRNA (*n* = 3). **b** Contour plots of a representative experiment on HeLa cells transfected with increasing concentrations of eGFP-mRNA

Next, we evaluated the effect of increasing the mRNA concentration (0.3, 0.9, and 1.5 µM). As can be seen from the flow cytometry data in Fig. 4a, the percentage of eGFP-positive cells increased for higher mRNA concentrations, reaching up to 38% eGFP-positive cells for 1.5 µM mRNA. This trend is furthermore illustrated in Fig. 4b, showing contour plots that display eGFP expression 24 h after a representative mRNA transfection experiment. Taken together, the results above provide a first proof-of-concept on the applicability of VNB photoporation for intracellular delivery of mRNA. Moreover, extensive optimization of different parameters related to the photoporation procedure allowed to obtain favorable mRNA transfection efficiencies of up to 38%.

Finally, to provide further proof that successful mRNA transfections are not limited to the eGFP-mRNA used so far, we proceeded with the transfection of murine MLKL (mixed lineage kinase domain-like)-encoding mRNA in B16F10 murine melanoma cells. MLKL is a known necroptosis executioner, *i.e.,* a type of immunogenic cell death, so that MLKL-mRNA transfection is expected to cause decreased cell viability [53]. Optimized VNB photoporation conditions for B16F10 cells were previously determined by our group, as reported by Van Hoecke & Raes et al*.* [43]. The results in Fig. S5 show that a significant drop in cell viability of 17% is obtained after transfection of MLKL-encoding mRNA in comparison with the VNB photoporation control without MLKL-mRNA. This level of mRNA transfection is in line with our expectations, given that eGFP-mRNA transfection efficiencies of ~ 16% are obtained in HeLa cells using similar VNB photoporation conditions (0.3 µM, 8 × 10^7^ AuNPs mL^−1^, 1.8 J cm^−2^). With these results, we demonstrated the applicability of the VNB photoporation technology for intracellular delivery of functional mRNA molecules as well.

### Transfection of Jurkat T Cells with eGFP-mRNA by VNB Photoporation

**Fig. 5:**
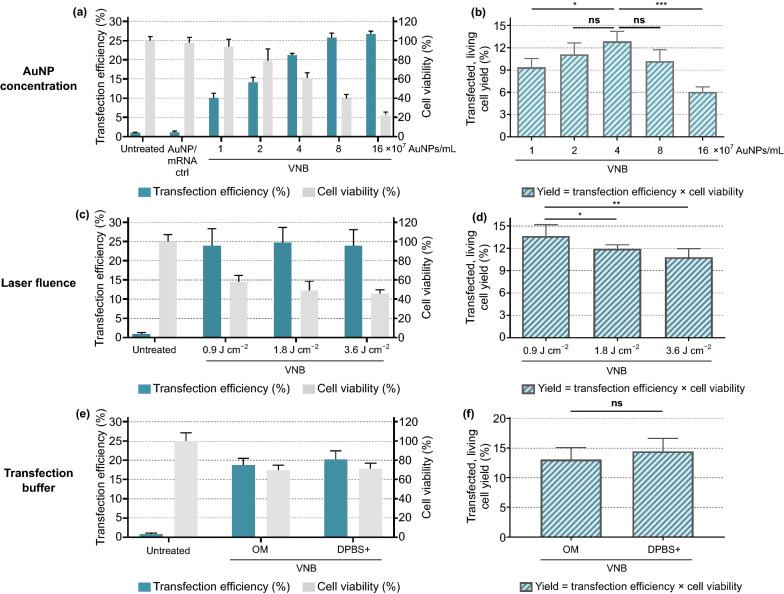
Optimization of gold nanoparticle (AuNP) concentration, laser fluence and transfection buffer for eGFP-mRNA transfection in Jurkat cells. Transfection efficiencies represent the percentage of eGFP-positive cells, cell viability values were calculated relatively to the untreated control and yields are calculated as the product of transfection efficiency and cell viability. **a, b** Screening for AuNP concentrations, using a fixed laser fluence of 1.8 J cm^*−*2^ (*n* = 3, one-way ANOVA with Dunnett’s multiple comparison test, *ns* = nonsignificant; *p < .05, ***p < .001). **c, d** Screening for laser fluences (in J cm^*−*2^), using a fixed AuNP concentration of 4 × 10^7^ AuNPs mL^−1^ (*n* = 3, one-way ANOVA with Dunnett’s multiple comparison test, *ns* = nonsignificant; *p < .05, **p < .01). **e, f** Comparison of Opti-MEM (OM) and DPBS + as transfection buffer, using a fixed AuNP concentration of 4 × 10^7^ AuNPs mL^−1^ and laser fluence of 0.9 J cm^−2^ (*n* = 3, unpaired Student’s *T* test, *ns* = nonsignificant)

The Jurkat E6-1 human leukemic T cell line was used here as a model for primary human T cells [47]. Analogous to the HeLa cell transfection experiments, different key parameters in the VNB photoporation procedure were first optimized for Jurkat cells (Fig. 5), i.e*.,* (1) AuNP concentration, (2) laser fluence and (3) transfection buffer.

Jurkat cells were first incubated for 30 min with increasing AuNP concentrations, ranging from 1 to 16 × 10^7^ AuNPs mL^−1^. Using confocal reflection microscopy, it was found that the corresponding number of cell-attached AuNPs ranged from ~ 1 to ~ 5 AuNP/cell (Fig. S6). After transfection by VNB photoporation with a laser fluence of 1.8 J cm^−2^, again an increasing percentage of eGFP-positive cells was obtained for increasing AuNP concentrations, with a concomitant decrease in cell viability (Fig. 5a). Eventually aimed at producing therapeutic engineered patient-derived T cells, in this case it is of interest to re-express these data as the percentage of transfected living cells. Indeed, limited T cell numbers are typically collected from profoundly lymphopenic patients owing to multiple previous rounds of cancer treatment, highlighting the need to maximize the production yield of viable, engineered T cells. As the data show in Fig. 5b, an optimum is found for 4 × 10^7^ AuNPs mL^−1^ (~ 2 AuNPs/cell) at which ~ 13% of the initial cell population is viable and transfected. In the next section, we will put these results in perspective against transfection by electroporation.

As a next step, different laser fluences were tested using a fixed AuNP concentration of 4 × 10^7^ AuNPs mL^−1^. eGFP expression was evaluated both qualitatively by confocal microscopy (Fig. S7) and quantitatively by flow cytometry (Fig. 5c, d). The percentage of positive cells did not increase with the laser fluence, but cell viability did decrease slightly (Fig. 5c). As a result, the best yield of living and transfected cells (~ 14%) was obtained for the lowest laser fluence of 0.9 J cm^−2^ (Fig. 5d). When evaluating the effect of different transfection buffers, contrary to HeLa cells, DPBS+ did not enhance eGFP-mRNA transfection of Jurkat cells (Fig. 5e, f). Therefore, we chose to continue further experiments on Jurkat cells using Opti-MEM as transfection buffer. These optimized conditions were furthermore shown to enable effective Luc mRNA transfection of Jurkat cells (Fig. S8).

**Fig. 6:**
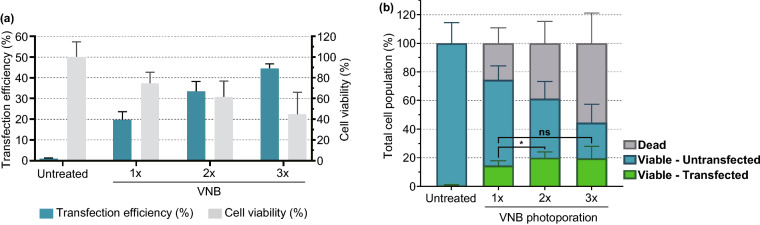
Effect of multiple consecutive VNB photoporation treatments on mRNA transfection efficiency of Jurkat cells. **a** Transfection efficiencies represent the percentage of eGFP-positive cells, and cell viability values were calculated relatively to the untreated control. **b** Viable/transfected, viable/untransfected and dead cell populations were calculated for the different conditions (*n* ≥ 3, One-way ANOVA with Tukey’s multiple comparison test, *p < .05, *ns* = nonsignificant)

Until this point, the most optimal conditions has led to 75% viable Jurkat cells of which 20% are transfected with eGFP-mRNA. This means that after applying one time the photoporation procedure, there remain still 60% Jurkat cells that are alive but untransfected. As such, it is of interest to try to repeat the photoporation procedure to see whether that can further enhance the final yield of living transfected cells. Figure 6 shows the results for 1× , 2×  and 3× photoporation of Jurkat cells. Between each of the procedures, cells were allowed to recover for 30 min, after which cells were again incubated with AuNP for 30 min and washed with Opti-MEM before photoporating again with eGFP-mRNA. After 2× photoporation, there remained 61% viable cells, of which now 33% are positive for eGFP. Repeating photoporation for a third time led to 45% viable cells, of which 45% was positive for eGFP. These results are summarized in Fig. 6b, showing for each repetition the fraction of nonviable (grey), viable untransfected (blue) and viable transfected (green) cells. Repeating photoporation two times increased the transfected cell yield significantly to 20% (*p* < 0.05). Repeating photoporation a third time did not produce a net beneficial effect as the increase in the number of transfected living cells is compensated for by an increase in cell death as well.

### VNB Photoporation Produces More Living mRNA-Transfected Jurkat Cells than Nucleofection

**Fig. 7:**
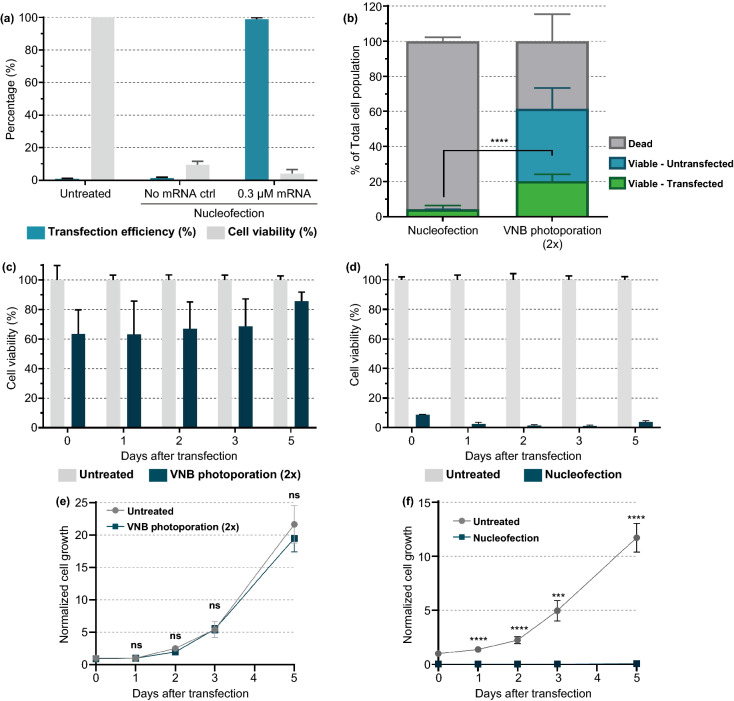
Comparison of nucleofection and VNB photoporation for mRNA transfection in Jurkat cells. **a** Jurkat cells were transfected with eGFP-mRNA (0.3 µM) by nucleofection according to the manufacturer instructions. The transfection efficiency (i.e., % eGFP-positive cells) and cell viability by a CellTiter-Glo assay were determined 24 h post-transfection (*n* = 3). **b** Comparison of nucleofection and VNB photoporation in terms of viable, transfected cell yield (*n* ≥ 3, unpaired Student’s *T* test; ****p < .0001). **c, d** Cell viability after VNB photoporation (**c**) or nucleofection (**d**) was measured at different time points for 5 days post-transfection. On day 0, cell viability was determined 2 h post-transfection. Cell viability values were calculated relatively to the untreated control on each day (*n* = 2 × 3*)*. **e, f** Normalized cell growth after VNB photoporation (**e**) or nucleofection (**f**) was measured at different time points for 5 days post-transfection. The normalized cell growth values were calculated relatively to the untreated control at day 0. Data are represented as the mean ± SEM (*n* = 2 × 3, unpaired Student’s *T* tests; ns = nonsignificant, ***p < .001, ****p < .0001)

Electroporation is currently the most common nonviral technique for transfection of nucleic acids and ex vivo modification of T cells [9]. Having previously optimized the VNB photoporation procedure for mRNA transfection of Jurkat cells, we here compared our technology with nucleofection as a state-of-the-art commercial electroporation system (Fig. 7). Nucleofection of Jurkat cells was performed using the optimized protocol from the manufacturer (Pulse code: CL-120; SE cell line solution); 24 h after transfection, a drastic impact on cell viability was observed with only 4% viable cells (Fig. 7a), which is in concordance with several other studies on transfection of lymphocytes by electroporation [27, 28]. Nearly all of those (98%) were transfected with eGFP-mRNA, leading to final yield of 4% living transfected cells after electroporation (Fig. 7b). This is about 5 × less as what we obtained with the two times repeated photoporation procedure.

In addition to acute cytotoxicity shortly after transfection, nucleofection was previously shown to significantly impact on the long-term behavior of T cells [28]. For this, we here extended the comparison between VNB photoporation and nucleofection by follow-up of the cell viability (Fig. 7c, d) and cell proliferation (Fig. 7e, f) up to 5 days after Jurkat T cell transfection. While cell viability remained favorable (> 60%) for VNB photoporation 5 days post-transfection, no improvement in cell viability was observed for cells transfected by nucleofection. We furthermore found no significant difference in cell growth between the untreated cells and photoporated cells, whereas no sign of recovery was observable even 5 days post-nucleofection. Altogether, these data put VNB photoporation forward as a more gentle approach for mRNA transfection of T cells.

## Discussion

In the last few years, cell-based therapeutics such as CAR T cells have emerged as a very promising approach for the treatment for hematological malignancies [13]. In 2020, over 500 clinical trials employing CARs have been reported worldwide, which clearly highlights the enthusiasm for adoptive cell therapies [56]. The success of T cell-based therapies, however, strongly depends on the ability to engineer these immune cells [9, 57]. Viral vectors are currently the clinical and commercial standard for this purpose, but they face multiple issues such as immunogenicity, high cost and variable outcomes. Indeed, transduction efficiencies typically range from a few percentages to over 80% in reported clinical trials [58–60]. As a consequence, mRNA-based cell therapies have come up as a safer and cheaper alternative to viral transductions [2].

In this work, we report for the first time on the use of VNB photoporation as a promising physical technique for gentle but efficient mRNA transfections. In its most common implementation, VNB photoporation harnesses a combination of plasmonic gold nanoparticles attached to the cell membrane and laser irradiation to transiently generate membrane pores and enable intracellular delivery of macromolecules. An incubation step of 30 min was previously found convenient to get the AuNPs well positioned for VNB photoporation, being either endocytosed but still in close proximity of the cell membrane (e.g., HeLa) or adsorbed to the cell membrane (e.g., Jurkat) [38, 44]. This AuNP incubation alone did not cause any significant cytotoxicity, which is in line with previously reported work on comparable AuNPs showing no impairment of cell viability or long-term cell homeostasis [23, 38]. At first, we evaluated and systematically optimized the VNB photoporation procedure for transfection of mRNA in the adherent HeLa cell line as a proof-of-concept. Several characteristics of mRNA make its intracellular delivery challenging, including their relatively large size, strong negative charge and susceptibility to degradation by nucleases [17]. The latter was indeed something we encountered in our study as well. Even though nucleotide-modified mRNA was used, gel electrophoresis clearly showed rapid degradation of mRNA within a few minutes after addition to the cultured cells [61]. This prompted us to include an extra washing step to remove remaining serum nucleases, which could prevent mRNA degradation for at least 10 min. While this is still quite short, it is sufficient to carry out the photoporation procedure which only took ~ 3 min. Based on earlier reports in the literature on the influence of the transfection buffer [17, 62], we also tried out different buffers for photoporation. We found that the percentage of transfected HeLa cells could be increased by a factor of ~ 1.5 using DPBS+ (containing Ca^2+^ and Mg^2+^) as transfection buffer instead of Opti-MEM. While supplementation with Ca^2+^ has been suggested to influence membrane repair kinetics [17, 63], we rather hypothesize that Ca^2+^ and Mg^2+^ may bind to mRNA, resulting in a reduced electrostatic repulsion between the mRNA molecules and the cell membrane. This is because the same enhanced effect of DPBS + was not observed for mRNA transfection of Jurkat cells which indeed have a lower density of negatively charged glycosaminoglycans on their cell membrane [44, 64]. Analogous to other physical transfection approaches, VNB photoporation locally disturbs the integrity of the plasma membrane and allows direct access to the cell cytoplasm. Once membrane pores are formed, mRNA molecules have only a short period of time (seconds to minutes) to reach the cell cytoplasm before membrane integrity is restored. Translocation of mRNA molecules to the cell cytoplasm is mainly thought to occur by passive diffusion during the pore lifetime [31]. For this, higher concentrations of the mRNA molecules were thought to increase the probability of mRNA molecules reaching the cytoplasm. Indeed, the percentage eGFP-positive cells reached up to 38% when using an mRNA concentration of 1.5 µM.

In the field of T cell-based therapeutics, the Jurkat T cell line is a frequently used model for primary human T cells [47]. Jurkat cells are, for instance, routinely used for initial in vitro screenings of novel CAR or engineered T cell receptor designs. A method that enables efficient and quick screening of different CAR constructs, without the need for designing a new dedicated viral vector for each construct, is therefore highly desirable [48–52, 65]. As a consequence, we selected the Jurkat T cell line to deliver the proof-of-concept that photoporation holds promise for the production of engineered T cells by mRNA transfections. Considering that high levels of transfection efficiency and cell recovery are both essential in the manufacturing of clinical-grade adoptive T cells [9], we expressed our transfection data in terms of the percentage of transfected, living cells. We demonstrated that photoporation could produce 14% transfected, living Jurkat cells. In addition, we showed that repeating the photoporation procedure a second time increased the transfected, living cell yield further to 20%. Most notably, this was fivefold more than what was obtained with nucleofection as a state-of-the-art electroporation technology. This is primarily due to the vast difference in the level of cytotoxicity induced by both techniques. Indeed, 24 h after treatment the metabolic activity of electroporated Jurkat cells had dropped dramatically to only ~ 4%, while this remained over 60% after two consecutive photoporation treatments. These results are in line with previous work on siRNA transfection of murine T cells [32], where photoporation yielded three times more living transfected cells as compared to electroporation.

Although electroporation was previously proven successful for mRNA transfection of T cells with efficiencies > 90% [66–69], more recent studies have raised the striking issue of extremely high acute cytotoxicity [27, 28], as we also showed here. Moreover, we demonstrated that Jurkat cells did not recover even 5 days post-nucleofection, whereas the cells treated by VNB photoporation maintained their proliferative potential. Apart from acute cytotoxicity, loss of functionality and nonspecific and unintentional changes in the cellular phenotype have been reported before as disadvantages of electroporation [27, 28]. These unfavorable effects were also shown to negatively influence the survival and in vivo potency of T cells to suppress tumor growth [27, 70]. At the same time, injection of nonviable T cells upon adoptive cell transfer can elicit immune responses and promote toxicity in vivo [71]. Spurred by the positive findings in our study, it will therefore be of interest to investigate the use of VNB photoporation for mRNA transfection of primary human T cells and its influence on T cell homeostasis and therapeutic functionality.

## Conclusion

Gold nanoparticle-mediated VNB photoporation proves to be a promising approach for safe and efficient intracellular mRNA delivery in both adherent and suspension cells. After rigorous optimization of different parameters, a good balance between mRNA transfection efficiency and cell survival was obtained. Most importantly, comparison of VNB photoporation and electroporation for mRNA transfection of Jurkat T cells indicated a marked fivefold increase in the percentage of transfected living cells for photoporation. These results position the VNB photoporation technology as a promising, more gentle approach toward safe and efficient engineering of T cells.

## Electronic supplementary material

Below is the link to the electronic supplementary material.

Supplementary file 1 (PDF 465 kb)
