# Supplementary material for: Intracellular Delivery of mRNA in Adherent and Suspension Cells by Vapor Nanobubble Photoporation
Source: Nanomicro Lett. 2020 Sep 27;12:185. doi: 10.1007/s40820-020-00523-0 (PMC7770675; doi:10.1007/s40820-020-00523-0)
Supplement: Supplementary file 1 — (PDF 465 kb) [file 40820_2020_523_MOESM1_ESM.pdf]

Supporting Information for

Intracellular Delivery of mRNA in Adherent and Suspension Cells by Vapor Nanobubble Photoporation

Laurens Raes[1, 2], Stephan Stremersch[1, 2], Juan C. Fraire[1], Toon Brans[1, 2], Glenn Goetgeluk[2, 3], Stijn De Munter[2, 3], Lien Van Hoecke[2, 4, 5], Rein Verbeke[1,2], Jelter Van Hoeck[1, 2], Ranhua Xiong[1], Xavier Saelens[4, 6], Bart Vandekerckhove[2, 3], Stefaan De Smedt[1, 2], Koen Raemdonck[1, 2], and Kevin Braeckmans[1, 2, *]

[1]Laboratory of General Biochemistry & Physical Pharmacy, Ghent University, 9000 Ghent, Belgium

[2]Cancer Research Institute Ghent (CRIG), 9000 Ghent, Belgium

[3]Department of Diagnostic Sciences, Ghent University, 9000 Ghent, Belgium

[4]VIB-UGent Center for Medical Biotechnology, 9052 Ghent, Belgium

[5]Department of Biomedical Molecular Biology, Ghent University, 9000 Ghent, Belgium

[6]Department of Biochemistry and Microbiology, Ghent University, 9000 Ghent, Belgium

*Corresponding author. E-mail: Kevin.Braeckmans@UGent.be (Kevin Braeckmans)

Supplementary Figures

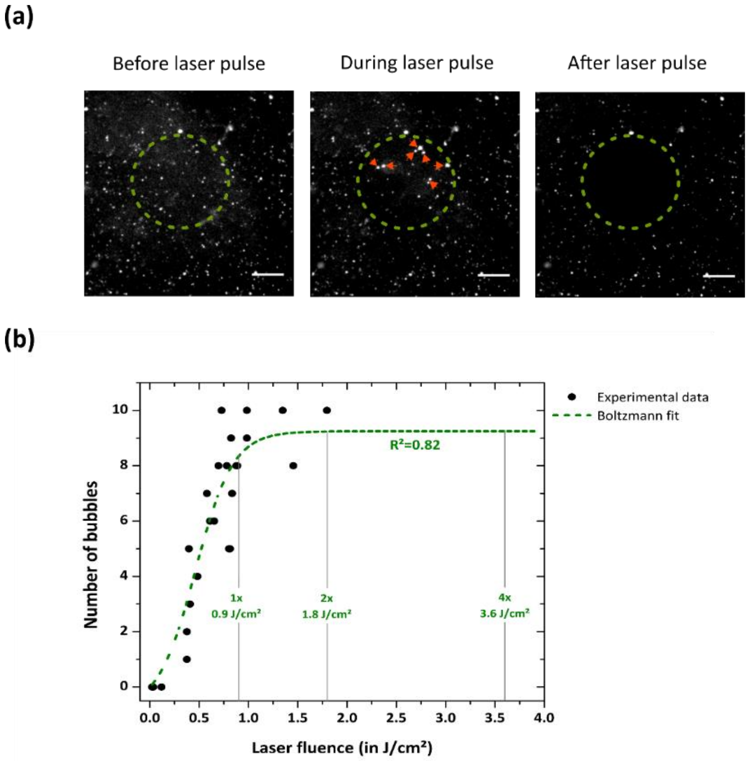

Fig. S1 Determination of the vapor nanobubble (VNB) generation threshold by dark field microscopy. **a** Dark field images of gold nanoparticles before, during and after laser irradiation (Scale bar=50 µm). The green dashed circle indicates the irradiated region with a diameter of 150 µm. During the laser pulse (I=0.88 J cm^{-2}), VNBs are visible as bright white spots because of an increase in light scattering, as indicated with orange arrows. **b** Number of visible VNBs was determined for increasing laser pulse fluences. From this plot, the VNB generation threshold is deduced by making use of a Boltzmann fit, which is defined as the laser fluence at which 90% of the asymptotic value of the Boltzmann fit is obtained (1×, 0.9 J cm^{-2})

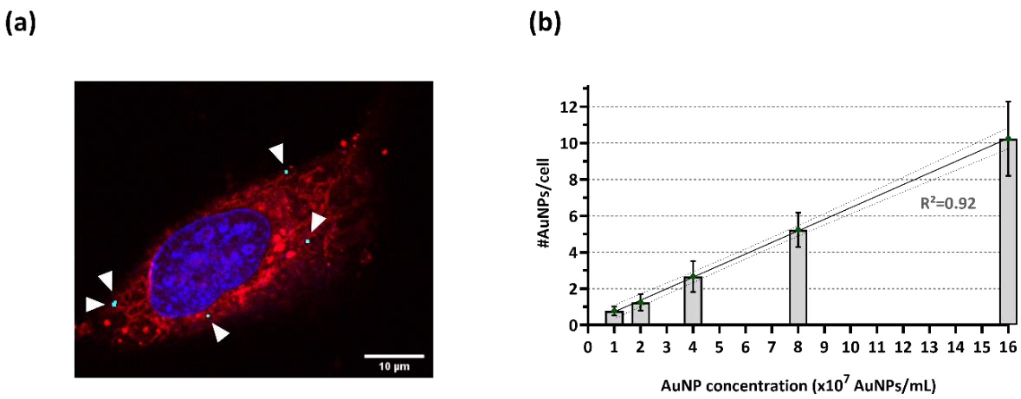

Fig. S2 Visualization and quantification of AuNP attachment to HeLa cells. **a** Visualization of AuNP adsorption to HeLa cells (AuNP concentration: 8x10^7 AuNPs/mL) by confocal reflection microscopy. The AuNPs are color coded in cyan blue and indicated with white arrow heads. Cytoplasm (red) and nuclei (blue) are stained with CellTrace Far red and Hoechst33342, respectively. **b** Quantification of the average number of AuNPs attached to HeLa cells for increasing AuNP concentrations (n=3 independent experiments, ≥50 cells/experiment)

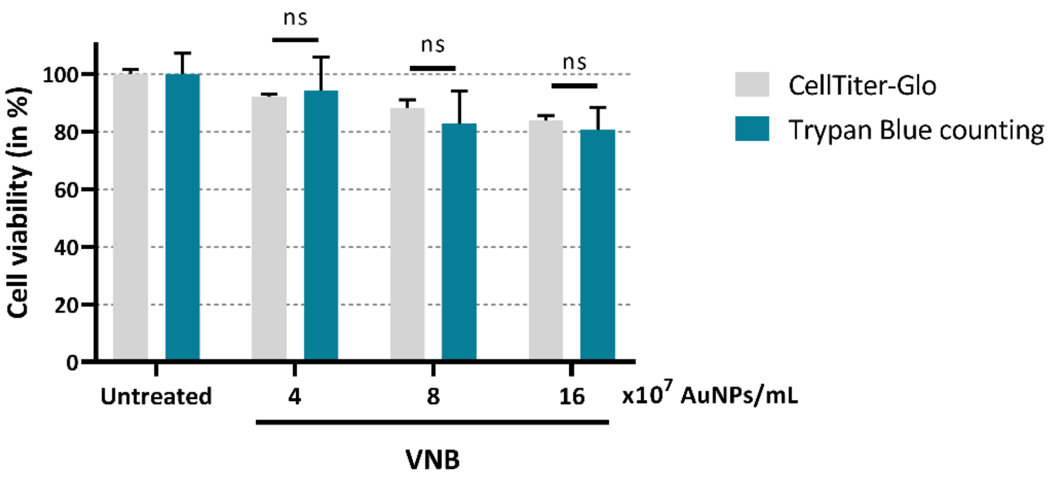

Fig. S3 Complementary analysis of HeLa cell viability after VNB photoporation by a CellTiter-Glo assay and cell counting. HeLa cells were photoporated with increasing AuNP concentrations using a fixed laser fluence of 1.8 J cm^{-2}. Cell viability values were determined with the CellTiter-Glo assay and trypan blue cell counting assay, expressed relatively to the untreated control (n=2). Individual paired student's T tests were performed to determine statistical differences between CellTiter-Glo and trypan blue cell counting for the different conditions (ns=non-significant)

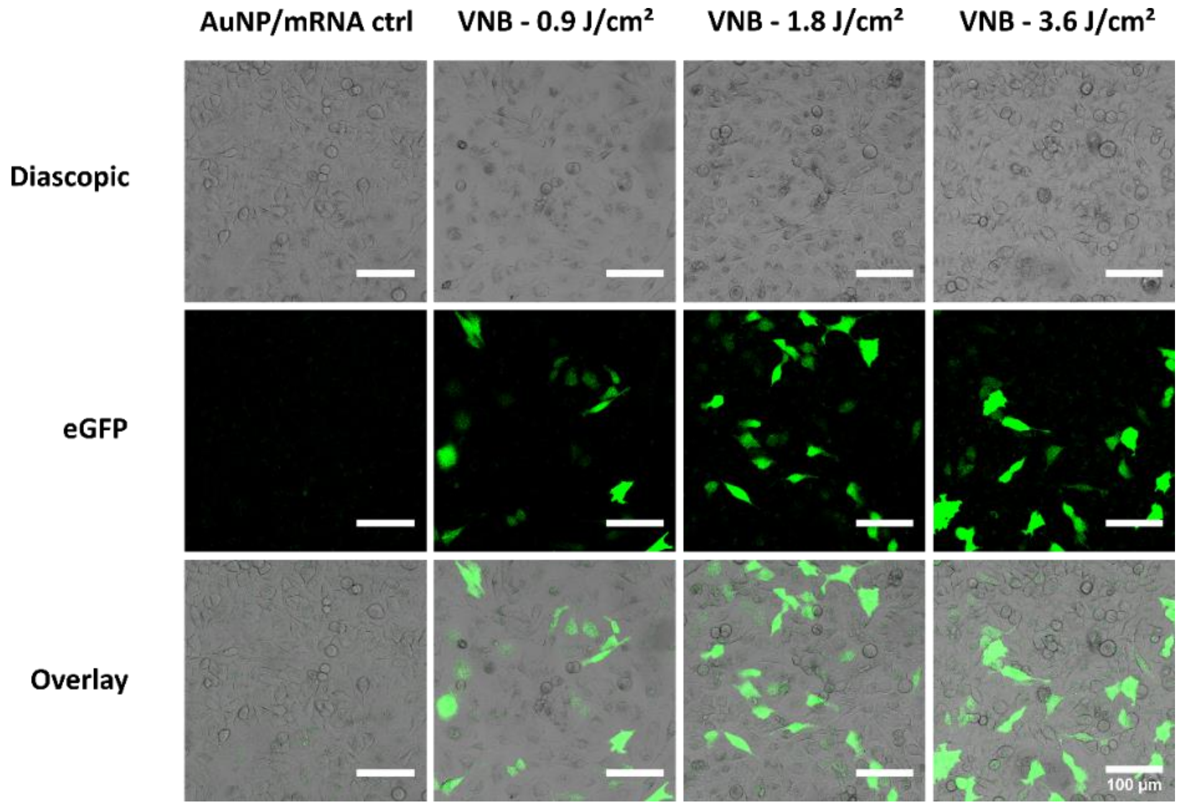

Fig. S4 Screening of different laser fluences for eGFP-mRNA transfection of HeLa cells. Confocal microscopy images of HeLa cells 24 h after transfection by VNB photoporation (AuNP concentration = 8×10^{7} AuNPs mL^{-1}) using increasing laser fluences as indicated in the figure (Scale bar=100 µm)

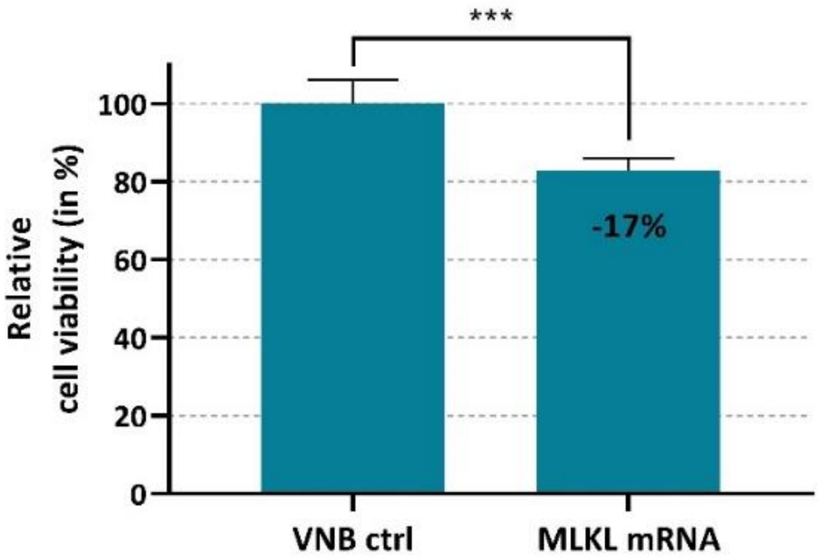

Fig. S5 MLKL-mRNA transfection in B16F10 murine melanoma cells by VNB photoporation. B16F10 cells were photoporated (8×10^{7} AuNPs mL^{-1}, 1.8 J cm^{-2}) in presence of 0.3 µM *in vitro* transcribed murine MLKL-mRNA. Induced cell death was quantified 18h after MLKL-mRNA transfection with the CellTiter-Glo viability assay and calculated relatively to the VNB photoporation control without mRNA (n=2). An unpaired student's T test was performed to determine the statistical difference (***p<.001)

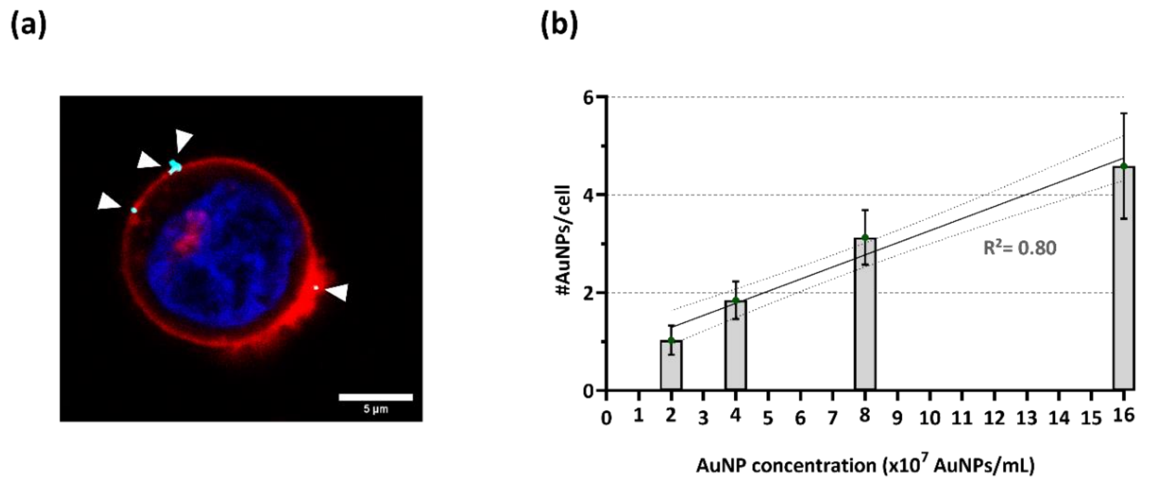

Fig. S6 Visualization and quantification of AuNP attachment to Jurkat cells. **a** Visualization of AuNP adsorption to Jurkat cells (AuNP concentration: 16×10^7 AuNPs mL^{-1}) by confocal reflection microscopy. The AuNPs are color coded in cyan blue and indicated with white arrow heads. Plasma membrane (red) and nuclei (blue) are stained with CellMask Deep Red and Hoechst33342, respectively. **b** Quantification of the average number of AuNPs attached to Jurkat cells for increasing AuNP concentrations (n=3 independent experiments, ≥150 cells/experiment)

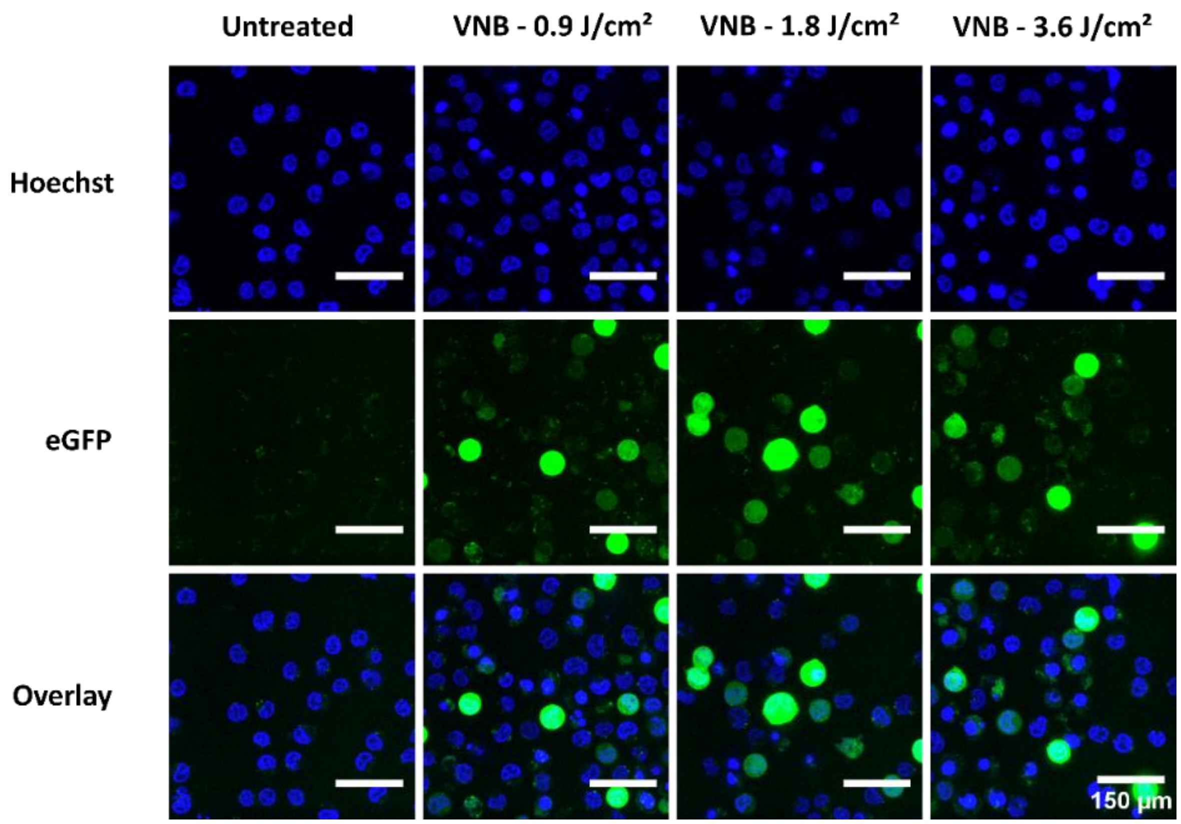

Fig. S7 Screening of different laser fluences for eGFP-mRNA transfection of Jurkat cells. Confocal microscopy images of Jurkat cells 24 h after transfection by VNB photoporation using increasing laser fluences (scale bar=150 µm)

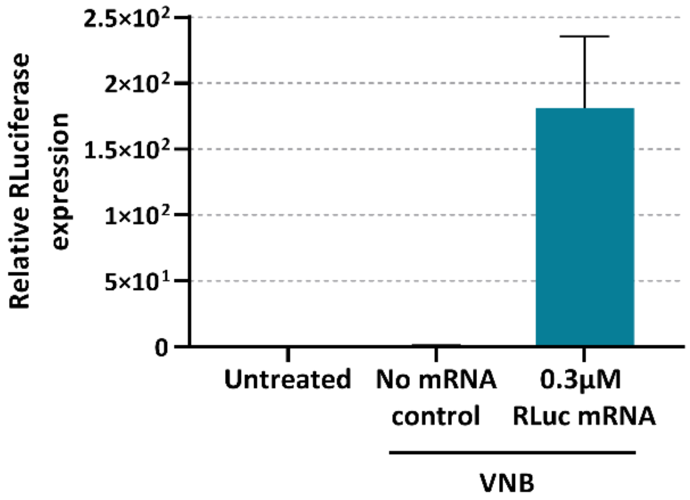

Fig. S8 RLuc mRNA transfection in Jurkat cells by VNB photoporation (0.3 µM, 4×10^7 AuNPs mL^{-1}, 0.9, J cm^{-2}, Opti-MEM). Background luminescence was substracted and expression data were normalized against the untreated control. VNB control was photoporated in absence of RLuc mRNA
